# Effects of different percentages of body weight-supported treadmill training in Parkinson's disease: a double-blind randomized controlled trial

**DOI:** 10.3906/sag-1812-57

**Published:** 2019-08-08

**Authors:** Tuğba ATAN, Özden ÖZYEMİŞCİ TAŞKIRAN, Ayşe BORA TOKÇAER, Gülçin KAYMAK KARATAŞ, Aslı KARAKUŞ ÇALIŞKAN, Belgin KARAOĞLAN

**Affiliations:** 1 Department of Physical Medicine and Rehabilitation, Faculty of Medicine, Hitit University, Çorum Turkey; 2 Department of Physical Medicine and Rehabilitation, Faculty of Medicine, Koç University, İstanbul Turkey; 3 Department of Neurology, Faculty of Medicine, Gazi University, Ankara Turkey; 4 Department of Physical Medicine and Rehabilitation, Faculty of Medicine, Gazi University, Ankara Turkey; 5 Department of Neurology, 29 Mayıs Public Hospital, Ankara Turkey; 6 Department of Physical Medicine and Rehabilitation, Faculty of Medicine, Gazi University, Ankara Turkey

**Keywords:** Parkinson's disease, gait, balance, treadmill training, fatigue

## Abstract

**Background/aim:**

Body weight-supported treadmill training (BWSTT) might have greater effects than conventional treadmill training (TT) in neurological diseases such as Parkinson's disease (PD). The aim of this study was to evaluate the effects of different percentages of BWSTT on gait, balance, quality of life, and fatigue in PD.

**Materials and methods:**

Thirty-five patients with moderate to advanced PD were randomized into three BWSTT groups according to the supported percentage of body weight: 0% BWSTT (control group; unsupported TT), 10% BWSTT, or 20% BWSTT. Five patients were excluded due to early discharge and 30 patients completed BWSTT sessions lasting 30 min, 5 days a week, for 6 weeks during their inpatient rehabilitation stay. The primary outcome was 6-min walk distance (6MWD). Secondary outcomes were Unified Parkinson's Disease Rating Scale (UPDRS), Berg Balance Scale (BBS), Nottingham Health Profile (NHP), Fatigue Impact Scale, and Fatigue Severity Scale scores. Measurements were performed before and after the training.

**Results:**

The unsupported TT group demonstrated no significant improvement in the outcome measures after a 6-week training except for BBS and NHP emotional subscores. Moreover, the NHP pain subscore increased in the unsupported TT group after training. The 10% and 20% supported BWSTT groups demonstrated significant improvements in 6MWD (P = 0.004 and P < 0.001, respectively), UPDRS - motor score (P = 0.012 and P = 0.005, respectively), NHP pain subscore (P = 0.003 and P = 0.002, respectively), and fatigue (P = 0.005 for both) after training. The 20% BWSTT provided the highest improvement in balance among the three groups (P < 0.001) and greater relief of fatigue than 10% BWSTT (P = 0.002).

**Conclusion:**

Six weeks of BWSTT improved walking distance and balance ability, relieved fatigue, and additionally reduced pain in patients with moderate to advanced PD.

## 1. Introduction

Parkinson's disease (PD) is a progressive and degenerative disorder characterized by an inadequate production of dopamine due to pathology in the substantia nigra. Rigidity, bradykinesia, and postural instability are the cardinal features that lead to gait impairment and functional limitations [1]. Gait and balance impairments are important determinants of disability and quality of life in PD [2]. Overall, fatigue is one of the most common and disabling nonmotor symptoms and can be seen at all stages of the disease [3].

Aerobic training with treadmill training (TT) is effective in improving the gait, balance, and quality of life and relieving fatigue in subjects with PD [4,5]. Body weight-supported treadmill training (BWSTT) allows safe walking practice by supporting a portion of the body weight mechanically and stimulates activity-dependent neural plasticity [6]. Furthermore, physical performance and aerobic activities can be performed at higher intensities when the body weight is partially supported during walking compared to conventional TT [7]. This is especially beneficial in the rehabilitation of neurologically impaired subjects, such as PD patients. Ganesan et al. showed that BWSTT had greater improvements of disability and gait parameters than ground walk training in PD. However, since the control group was not trained on a treadmill wearing a harness without partial body weight support, study participants were not blinded to the study. They also included only 20% supported BWSTT in the intervention group. Hence, it is not possible to interpret the effect of BWSTT over TT or the effects of different amounts of body weight support on clinical and gait performance. In that study, the effects of BWSTT on balance, quality of life, and fatigue were not investigated as outcome measures, either [8].

The primary hypothesis of this study is that 20% supported BWSTT will lead to greater improvements in gait performance than 10% supported BWSTT or unsupported TT in PD. The second hypothesis is that BWSTT will provide significant improvements in balance, disability, quality of life, and fatigue. To test these hypotheses, it is aimed to assess the effects of 20%, 10%, and 0% BWSTT on gait as well as balance, quality of life, and fatigue in subjects with PD.

## 2. Materials and methods

This randomized controlled double-blind study was approved by the Ethics Committee of the Gazi University Medical Faculty (No: 2010-171) and registered in the Clinical Trials database (NCT03799887). Participants were fully informed about the procedures and written consent was obtained.

### 2.1. Participants

Participants who were diagnosed with idiopathic PD according to the UK Brain Bank criteria were recruited from the Movement Disorders Outpatient Clinic of the Gazi University Neurology Department. Patients with moderate to advanced disease (Hoehn and Yahr stage 2–4) with stable doses of dopaminomimetics for at least 4 weeks and who were able to walk with or without assistive devices were included. Exclusion criteria were cardiovascular, inflammatory, musculoskeletal, or cognitive problems (Mini Mental Status Examination (MMSE) score of less than 26) that could prevent their participation in the training program. Participants were hospitalized in the inpatient rehabilitation unit of the Gazi University Physical Medicine and Rehabilitation Department.

### 2.2. Measurements

Demographic features, clinical parameters, Hoehn and Yahr Stages, MMSE scores, and medications were recorded. Hoehn and Yahr staging measures the severity of PD in stages 0 through 5. Higher stages mean more advanced disease. Stage 0 means no findings of the disease and stage 5 means the most advanced disease in which the patient is wheelchair-bound or bedridden unless aided. The Turkish version of the MMSE was used to assess cognitive status [9].

The following measurements were performed before and after the training program by a blinded physiatrist and neurologist at the same time of the day. Medications for PD were kept at stable doses during the study and the outcome measurements and interventions were performed during “on” periods.

### 2.3. Primary outcome measurement 

The 6-min walk test (6MWT) is a submaximal exercise test usually corresponding to 80% of a subject's maximum heart rate and is used to assess functional capacity and treatment response [10,11]. The patients were asked to walk as long as possible for 6 min on 30 m of marked and flat ground at a self-selected speed. Standard instructions were used and ambulatory devices were permitted. Distance walked in 6 min (6MWD) was recorded. Healthy people have an average walking distance of 500–700 m [12].  

### 2.4. Secondary outcome measurements 

The Berg Balance Scale (BBS) contains 14 items. Each item is scored from 0 (total inability to perform the activity) to 4 points (ability to perform the activity independently). Higher scores indicate a better balance ability [13]. The Turkish version was used [14].

The Unified Parkinson's Disease Rating Scale (UPDRS) is used to assess disability in PD. It consists of four main parts (totally 183 points): mentation, behavior, and mood (UPDRS I: 16 points); activities of daily living (UPDRS II: 52 points); motor examination (UPDRS III: 92 points); and treatment complications (UPDRS IV: 23 points). Higher scores indicate worse clinical disease. UPDRS I, II, and III were used in this study. UPDRS III in particular helps to monitor treatment and measure the effectiveness of treatment. Speech, facial expression, tremor at rest, action tremor of hands, rigidity, finger taps, hand grips, pronation-supination movements of hands, leg agility, rising from chair, posture, gait, postural stability, and body bradykinesia were evaluated. For this section each item is scored from 0 (normal) to 4 (unable to do it). Higher scores indicate worse clinical disease. The UPDRS was administered by a neurologist specialized in movement disorders.

The Turkish version of Nottingham Health Profile (NHP) was used to assess health-related quality of life [15]. It contains 38 items that address pain, physical mobility, emotional reactions, energy, social isolation, and sleep dimensions. Higher scores indicate worse quality of life.

The Fatigue Impact Scale (FIS) assesses the cognitive, physical, and social effects of fatigue during the last week in a 40-item questionnaire (0 = no problem, 4 = maximum problem). The total score ranges from 0 to 160. Higher scores reflect a higher degree of fatigue [16]. The validity and reliability of the Turkish version was demonstrated [17]. 

The Fatigue Severity Scale (FSS) assesses the severity of fatigue during the last week in a 9-item questionnaire (1 = strongly disagree, 7 = strongly agree). Total score ranges from 9 to 63, with higher scores representing greater fatigue [18]. The validity and reliability of the Turkish version was shown [19].

### 2.5. Interventions

All participants received 30 min of conventional rehabilitation including range of motion, stretching, strengthening, and balance exercises followed by 30 min of BWSTT performed on a BWSTT unit (Biodex Medical Unweighing System, Model 945-480 (serial no: 04111171), Shirley, NY, USA), 5 days a week, for 6 weeks. Each BWSTT session consisted of a 5-min warm-up and cool-down period and was intended to include 25 min of submaximal aerobic exercise. Exercise intensity was adjusted according to 6MWD. Heart rate achieved at the end of the 6MWT was regarded as the target BWSTT heart rate [20]. This exercise intensity was submaximal and the heart rate and blood pressure were measured during training sessions, and treadmill speed was tailored to reach the target HR. Maintenance of the heart rate during training sessions was important both for providing standard exercise intensity for each patient and for maintaining cardiovascular safety to prevent unwanted cardiac events, especially in patients with cardiovascular disease and hypertension. Feedback related to speed of gait, symmetry of step length, or posture was given to the patients by the physiotherapists. Amount of body weight support applied during BWSTT was set according to the randomization. 

#### 2.5.1. Randomization

Participants were randomized by a computer program into three groups according to the supported percentage of body weight: 0% BWSTT (control group; unsupported TT), 10% BWSTT, or 20% BWSTT after age, sex, and stage of disease matched blocks were constructed. A second physiatrist who was not involved in the assessment of study outcome measures performed the randomization and prescribed the individualized rehabilitation program accordingly. This prescribed rehabilitation program was administered by two physiotherapists who were told not to mention the treatment allocation to the participants and the blinded physiatrist and neurologist. Before discharge from the hospital, all outcome assessments were repeated by the same blinded physiatrist and neurologist.

The power of this study was calculated as 0.84 with 10 patients in each group, with noncentrality parameter of 11.98 and type-I error rate of 0.05. The schematic flow diagram of the study is presented in the Figure. Thirty-five patients with idiopathic PD were included in the study. Five patients were discharged early and lost to follow-up, leaving 10 patients in each group.

**Figure:**
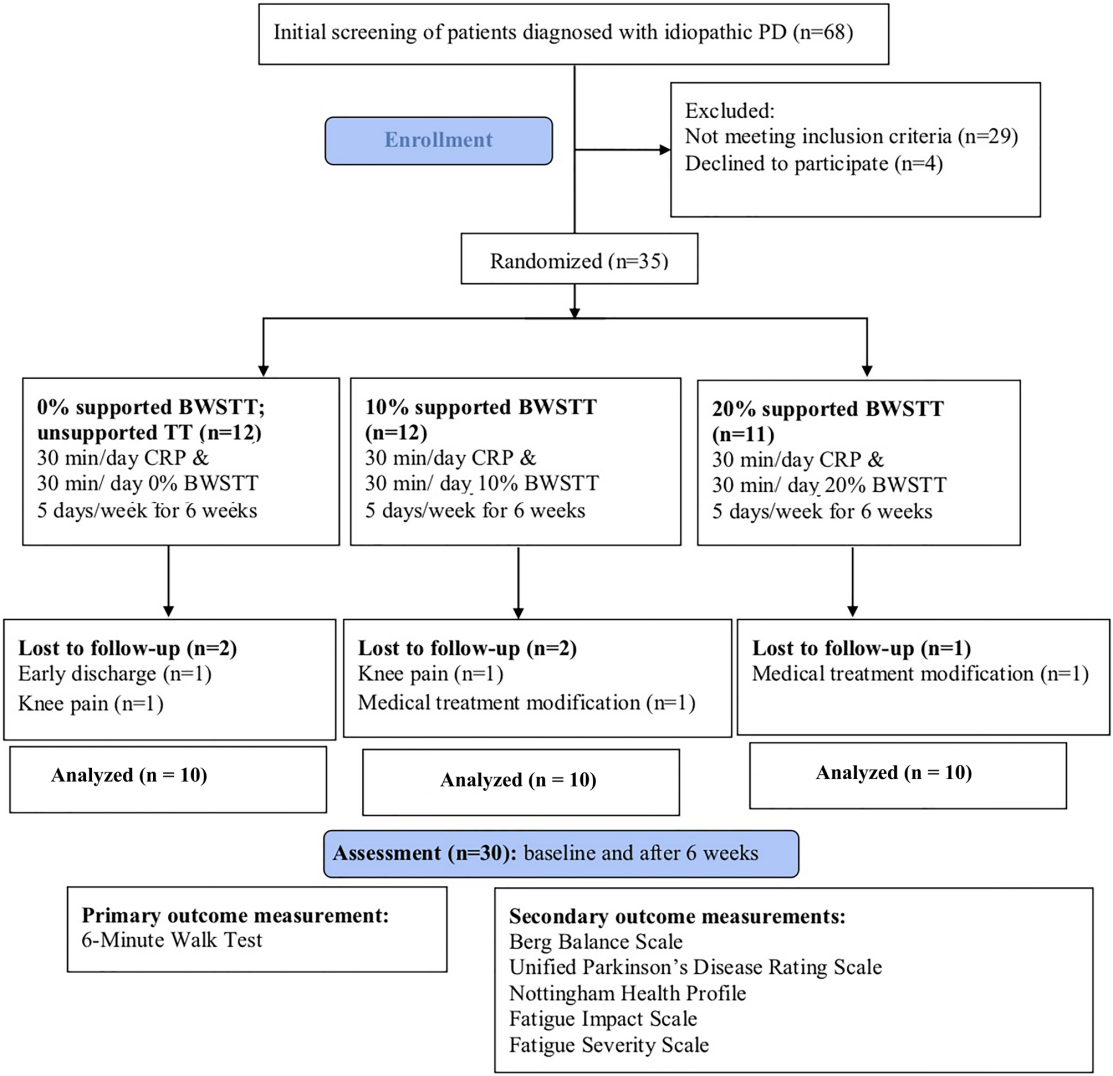
The schematic flow diagram of the study design. PD: Parkinson’s disease, BWSTT: body weight-supported treadmill training, CRP: conventional rehabilitation program, 6MWT: 6-min walk test, BBS: Berg Balance Scale, UPDRS: Unified Parkinson’s Disease Rating Scale, NHP: Nottingham Health Profile, FIS: Fatigue Impact Scale, FSS: Fatigue Severity Scale.

### 2.6. Statistical analysis 

Demographic data and clinical features are presented as mean and standard deviation (SD). A design of 3 (groups) × 2 (times – baseline and 6th week) was used. Baseline measurements among groups were compared using one-way analysis of variance (ANOVA). The paired samples t-test was used to assess the differences between pre- and posttraining within each group. Statistically different parameters were analyzed by Mann–Whitney U test to verify the group the difference originated from. A Scheffe multiple comparison test was used after ANOVA. Effect size (ES) was used to compare differences in the evaluation of exercise training among groups. ES was used to evaluate the amount of change in outcome measurements in each intervention group. ES was identified as 0.2–0.5 = small, 0.5–0.8 = moderate, and >0.8 = large. Statistical analysis was performed with SPSS 21.0 for Windows (IBM Corp., Armonk, NY, USA) and P ≤ 0.05 was considered a statistically significant difference. 

## 3. Results

Age, sex, height, weight, body mass index, duration and severity of PD, levodopa equivalent doses, and MMSE scores did not differ (all P > 0.05) among the three groups (Table 1).

**Table 1:** Demographic and clinical features of the groups.

		0% unsupported(n = 10)	10% supported (n = 10)	20% supported(n = 10)	P
Age, years, mean (SD)		69.7 (8.0)	72.2 (7.9)	68.6 (8.2)	0.596
Women, n (%)		7 (70%)	6 (60%)	6 (60%)	0.886
Height, cm, mean (SD)		164.1 (7.7)	162.6 (9.6)	162.2 (5.4)	0.847
Weight, kg, mean (SD)		73.6 (13.7)	77.1 (9.7)	72.2 (11.3)	0.632
BMI, kg/m², mean (SD)		27.62 (6.74)	29.17 (3.04)	27.58 (4.56)	0.724
Duration of disease, years, mean (SD)		5.6 (5.3)	9.8 (9.0)	7.6 (6.4)	0.347
Hoehn and Yahr	Stage 2, n (%)	5 (50%)	3 (30%)	4 (40%)	0.139	Stage 3, n (%)	4 (40%)	6 (60%)	5 (50%)	Stage 4, n (%)	1 (10%)	1(10%)	1(10%)
	Levodopa equivalent dose, mg, mean (SD)		698.1 (207.2)	696.5 (195.5)		884.0 (253.6)	0.110
	MMSE score, mean (SD)		28.4 (1.8)	28.8 (1.6)		28.3 (1.8)	0.796
Comorbidities, n (%)					
HypertensionDiabetes mellitusCardiovascular diseaseAnemiaOsteoarthritis		7 (70%)	5 (50%)	3 (30%)	0.218	2 (20%)	4 (40%)	2 (20%)	0.534	3 (30%)	1 (10%)	2 (20%)	0.563	0 (0%)	1 (10%)	3 (30%)	0.142	5 (50%)	5 (50%)	6 (60%)	0.886
		Usage of ambulatory assistive device, n (%)		4 (40%)	4 (40%)	2(20%)	0.576

BMI: Body mass index, MMSE: Mini-Mental State Examination, SD: standard deviation.

Thirty participants completed the 6-week training program, which was well tolerated, and no adverse events were observed except for knee pain. Adherence to the training program was similar among the groups (Table 2). 

**Table 2:** Features of completed training sessions in different body weight-supported treadmill training groups.

	0% unsupported,mean (SD)	10% supported,mean (SD)	20% supported,mean (SD)	P
Number of sessions	25.3 (5.1)	21.9 (6.8)	24.2 (8.2)	0.537
Duration of sessions, min	73.5 (13.1)	61.0 (3.2)	69.5 (9.8)	0.223
Total duration of aerobic exercise, min	25.5 (5.9)	22.5 (5.4)	23.5 (5.2)	0.481
Duration of submaximal aerobic exercise, min	12.7 (2.9)	11.2 (2.7)	11.7 (2.6)	0.481

SD: Standard deviation, min: minutes.

The unsupported TT group demonstrated no significant improvement in the outcome measures after training except for BBS and NHP emotional subscores (Table 3).

**Table 3:** Walking distance, balance, UPDRS, NHP, and fatigue scores before and after training.

	0% unsupportedMean (SD)	10% supportedMean (SD)	20% supportedMean (SD)	P1P2	Effectsize (d)
6MWD, mBasal6th week	206.6 (111.4)222.5 (108.8)	188.6 (106.4)272.7 (124.3)*	164 (59.8)374.5 (130.9)*	0.6110.059	–0.49
BBSBasal6th week	35.8 (7.3)40.9 (7.1)*	36.5 (5.0)45.5 (7.5)*	32.2 (7.5)51.7 (2.6)*	0.3210.004	–1.84
UPDRS IBasal6th weekUPDRS IIBasal6th weekUPDRS IIIBasal6th week	1.9 (2.6)1.7 (2.1)13.5 (6.5)11.4 (5.8)	0.9 (1.0)0.9 (1.1)10.5 (6.0)11.8 (6.4)	1.6 (1.8)1.6 (1.6)11.4 (5.3)10.4 (5.0)	0.5070.4970.5220.855	0.030.10	20.9 (9.3)17.8 (5.7)	19.2 (7.8)13.3 (5.6)*	25.8 (7.1)9.7 (4.0)*	0.1870.067	1.15
	NHP painBasal6th weekNHP emotionalBasal6th weekNHP energyBasal6th weekNHP physicalBasal6th weekNHP social isolationBasal6th weekNHP sleepBasal6th week	37.5 (34.4)48.8 (32.5)*38.9 (37.1)27.8 (30.6)*	50.0 (33.3)30 (35.0)*40.0 (35.2)31.1 (33.9)	53.8 (27.0)6.3 (19.8)*21.1 (24.3)12.2 (17.7)*	0.4970.0130.3640.297	0.570.31	60.0 (41.0)49.9 (39.3)57.5 (27.8)56.3 (30.8)	67.5 (38.6)43.4 (31.6)*60.0 (18.4)45.0 (14.7)*	62.5 (25.8)3.3 (10.5)*55.0 (18.8)5.0 (8.7)*	0.8930.0030.880<0.001	0.880.85	24.0 (33.7)16.0 (26.3)46.0 (32.7)46.0 (37.8)	20.0 (32.6)18.0 (30.5)48.0 (39.1)40.0 (36.5)	16.0 (18.4)6.0 (9.7)36.0 (33.7)33.5 (29.1)	0.8290.4950.7210.725	0.250.10
FISBasal6th week		57.5 (32.4)52.4 (33.1)	62.5 (30.5)46.1 (28.3)*	107.3 (24.1)19.7 (10.1)*	0.0080.061	1.11
FSSBasal6th week		5.0 (1.6)4.8 (1.6)	5.2 (1.8)3.7 (1.6)*	5.5 (1.2)1.9 (0.5)*	0.763<0.001	1.05

P1: P-value for basal measurements among the three groups. P2: P-value for the measurements at the 6th week among the three groups. *: P < 0.05 for pre- and posttraining measurements for each group. 6MWD: 6-min walk distance, BBS: Berg Balance Scale, UPDRS: Unified Parkinson’s Disease Rating Scale, NHP: Nottingham Health Profile, FIS: Fatigue Impact Scale, FSS: Fatigue Severity Scale, m: meters, SD: standard deviation.

After training, the 6MWD improved significantly in the 10% and 20% supported groups. The 20% supported group achieved the greatest 6MWD at the 6th week among the three groups; however, this did not reach statistical significance. 

All groups showed significant increases in BBS after training compared to baseline (P = 0.008, P = 0.011, and P = 0.005, respectively). The difference in the 6th week BBS scores among groups was significant, originating from the 20% supported group (P < 0.001). 

After training, the UPDRS III scores were significantly decreased in the 10% and 20% supported groups (P = 0.012 and P = 0.005, respectively). The 20% supported group demonstrated the greatest amount of reduction in UPDRS III among the three groups, which was not statistically significant.

Pain, energy, and physical subscores of NHP were significantly different among groups. Pain subscores at the 6th week increased significantly in the unsupported TT group (P = 0.019), whereas they decreased in the 10% and 20% supported groups (P = 0.003 and P = 0.002, respectively) compared to baseline. There were significant improvements in the 10% and 20% supported groups in energy (P = 0.004 and P < 0.001, respectively) and physical subscores (P = 0.003 and P = 0.002, respectively).

After training, the 10% and 20% supported groups showed significant improvements in both FIS and FSS scores (P = 0.005, both groups for each score). The 20% supported group showed the greatest amount of reduction in FIS and FSS scores after training but only the difference in FSS reached statistical significance (P = 0.002).

## 4. Discussion

The findings of this study supported our primary hypothesis that BWSTT would improve gait performance in subjects with PD. Both the 10% and 20% supported BWSTT groups demonstrated improvements in walking distance, whereas unsupported TT did not show significant improvement. Regarding the second hypothesis, balance improvement was greatest in the 20% supported BWSTT group and UPDRS motor scores improved in both supported BWSTT groups, but did not improve in unsupported TT. The 6-week BWSTT also improved the quality of life and relieved fatigue compared with unsupported TT in PD.

Improvements observed in gait and UPDRS motor scores after BWSTT are consistent with previous PD studies [7,8,21–23]. In the literature, 20% is the most widely used percentage of unweighing in BWSTT studies, as individuals with PD reported that 20% body weight support was the most comfortable among 0%, 10%, 20%, and 30% unweighted supports [23]. Researchers used either 20% [7,8] or a combination of 20% and 10% [22–24] unweighing in their BWSTT protocols and compared BWSTT to overground gait training [7,8,24] or traditional rehabilitation programs [22,23]. None of these studies performed blinding of the participants. Our study is unique in comparing two different percentages of BWSTT (10% and 20%) to an unsupported TT group and providing blindness of the study participants. 

On the other hand, a recent study did not prove a superior effect of BWSTT over conventional TT and showed that they both improved gait, balance, and disability in PD [24]. That study suggested that BWSTT might be preferred in advanced PD patients with severe postural instability, impaired balance, or orthostatic hypotension that would limit conventional TT. 

Only one study investigated the effect of BWSTT on balance in PD. Balance improved only in the BWSTT group (20% support) compared to either conventional ground gait training or the nonexercised control group. None of the groups were trained with balance exercises [7]. Contrary to that study, a conventional rehabilitation program including balance training was administered to each group in our study with the concern of ethical issues. This might explain the increase in BBS scores in all the groups after training, which was different from the study of Ganesan et al. However, the greatest improvement was observed in the 20% BWSTT group in our study, indicating the additional effect of BWSTT on balance performance.

Regarding the quality of life, none of the previous studies investigated the effect of BWSTT in PD. In our study, we observed that only the supported BWSTT (10% and 20%) groups, not the unsupported TT group, showed improvements in energy and physical subscores of NHP after training. Interestingly, the pain subscore of NHP increased significantly in the unsupported TT group while it decreased in both supported groups. We suggest that BWSTT might protect the joints by decreasing their loading and thus provide movement with less pain. This may be important in PD as subjects with moderate to advanced disease have higher rates of pain, which may be related to concomitant osteoarthritis [25,26]. Supporting our findings, in a previous study of knee osteoarthritis [27], pain reduction was achieved after 12 weeks of lower body positive pressure supported TT compared with full body weight TT.

Fatigue was found as the nonmotor symptom most strongly associated with the level of physical activity in PD [28]. Implementing physical activity, especially aerobic training, is shown to be highly effective to reduce fatigue in patients with other various medical conditions, including multiple sclerosis [29], systemic lupus erythematosus [30], and cancer [31]. Similarly, higher levels of physical activity are expected to have a beneficial effect on relieving fatigue in PD. Fatigue is reported by nearly half of patients with PD and a metaanalysis of only two studies showed no significant effect of aerobic exercise on the management of fatigue [32,33]. However, the effect of BWSTT on fatigue has not been examined. To our knowledge, this is the first study to investigate the effect of BWSTT on fatigue. We found that 6 weeks of BWSTT significantly reduced fatigue in PD compared with unsupported TT. We suggest that BWSTT might enhance longer durations of aerobic exercise by providing higher walking speed with lowered risk of falls and decreased burdens on the cardiopulmonary system [34] compared to full body weight walking. Higher pain levels in the unsupported TT group might provide an explanation for decreased tolerability of full body weight aerobic exercise, which limits its capability to increase the functional capacity and physical fitness of the patient and hence reduce fatigue. BWSTT might have promising results in the management of fatigue in people with PD. 

Importantly, BWSTT was well tolerated and participants reported few adverse events such as muscle or joint pain. Contrary to our observations, Berra et al. reported that four patients with chronic pain or anxiety could not tolerate BWSTT [24]. Although participants had moderate to advanced PD, adherence to the treatment was high in all groups. A previous study showed the effectiveness of an intensive inpatient rehabilitation with improved motor functions and Hoehn and Yahr Stages in advanced PD [35]. Inpatient stay during the rehabilitation program might play a role in achieving high rates of participation. 

As a limitation of this study, the follow-up period was short for understanding the maintenance of the training effects. Further studies are necessary to determine the long-term effects of BWSTT in subjects with PD.

In conclusion, a 6-week BWSTT program with 10% or 20% support improved walking distance, balance, UPDRS motor score, quality of life, and fatigue compared with unsupported TT in subjects with PD. The 20% BWSTT provided superior results in improving balance and fatigue. 
